# The Role of an Intraorganizational Digital Community in Shaping Nurses’ Professional Identities and Practice: Qualitative Interview Study

**DOI:** 10.2196/81765

**Published:** 2025-11-20

**Authors:** Etti Rosenberg, Stefan Cojocaru

**Affiliations:** 1 Department of Sociology, Social Work and Human Resources, Alexandru Ioan Cuza University, Bulevardul Carol I 11, Iași, 700506, Romania, 40 744788779

**Keywords:** social media, professional identity, intraorganizational digital community, nursing, organizational culture, self-efficacy, sense of community

## Abstract

**Background:**

In 2017, Israel’s health organizations established intraorganizational social media communities, believing that they would serve as a tool that would enable people to share experiences across regional boundaries. However, conducting preliminary studies and analyzing the findings to determine how they affected employees’ experience was never part of this effort.

**Objective:**

This study examined the impact of an intraorganizational digital community on nurses’ professional identities and practices within a large health care organization.

**Methods:**

Using a qualitative descriptive approach, semistructured interviews were conducted with 20 nurses from various specialties and regions participating in an intraorganizational nurses’ community on Facebook.

**Results:**

The findings showed that the intraorganizational community fostered a strong sense of belonging, emotional support, and professional development among its members. Participants talked about having a sense of community, much like being a member of a family, where they could confide in one another, ask for help and advice, and receive support. Enriched professional knowledge, self-efficacy, and pride in the nursing profession were all associated with active involvement in the community. The complex interactions of social media use in a hierarchical health care system were emphatically acknowledged by addressing challenges, including information overflow and concerns about sustaining a professional persona in a public digital domain.

**Conclusions:**

Overall, the study illustrated how crucial it is for health care organizations to actively manage potential negative consequences while using the benefits of intraorganizational digital networks, such as improving supportive relationships and ongoing shared learning. This study contributes to the growing body of knowledge regarding the crossroad between social media and health care, offering insights into developing strategies to promote a supportive and connected nursing workforce. The implications are particularly relevant for organizations seeking to strengthen nurse well-being and professional development through innovative digital tools. Future research should include quantitative studies to assess an intraorganizational platform’s influence on outcomes such as nurses’ sense of community, professional identity, self-efficacy retention, and job satisfaction.

## Introduction

### Evolution of Social Media in Health Care Organizations

Social media is reshaping health care organizations’ function and communication. Innovative digital platforms enable professionals to exchange information, share experiences, and connect with colleagues across geographic boundaries. This shift raises substantial questions about how these technologies affect nurses’ professional identity, daily practices, and sense of community within the workplace. Recent studies indicate that social media is changing health care workers’ communication dynamics by enhancing peer interaction and knowledge sharing. Increased use of social networks is associated with enhanced empowerment of both patients and health care professionals. Regarding patients, health-related digital communities enable individuals to interact with others facing similar conditions, access support communities that provide reliable medical information, and become more actively involved in their own care [1].

Internally, a digital community can help nurture a sense of community; promote professional networking; and exchange fruitful ideas, practices, and organizational professionalism. According to Chen and Wang [1], social media’s vast user base also offers valuable data for understanding behavioral and public health trends. The existing literature presents diverse perspectives on these innovations. Some studies suggest that social media can support collaborative research efforts and improve the understanding of health-related issues. These interactive platforms change how patients, health care professionals, and organizations communicate by fostering connections and facilitating information.

### Social Theory and the Digital Community

Traditional social science theories enhance our comprehension of digital nurse communities. Social identity theory [2] suggests that being part of a group enhances an individual’s self-image. In other words, belonging to a respected group (such as a professional community) can enhance an individual’s identity and pride. Engaging in a digital community can strengthen nurses’ professional identity by offering validation and a sense of belonging. The idea of digital communities by Rheingold [3] and the theory of communities of practice by Wenger [4] indicate that individuals who interact through digital platforms can cultivate shared values, norms, and knowledge, creating a unified social group even when they are not in the exact physical location.

The dramaturgical theory by Goffman [5] helps understand how people interact and express themselves online. The theory was applied to social networks such as Facebook by Merunková and Šlerka [6], who identified various self-presentation strategies (sharing personal stories, influencer style, valuable content, etc). Their study revealed that, similar to other users, nurses in a work-related digital community seeking to preserve their professional image may be mindful of what they post and consciously manage their online image and privacy.

Because of this awareness of the context and audience, interactions on a nurse’s online community are not completely spontaneous; instead, they are influenced by the need to maintain professional standards and an expectation for support. According to Goffman [5], nurses play roles in digital communities in ways similar to offline roles, balancing competence and truthfulness. According to theories of social identity and community of practice, an active intraorganizational social media community can help nurses develop a strong sense of shared identity. On the other hand, the dramaturgical theory by Goffman [5] serves as a reminder that role expectations and image maintenance still influence conduct in such a group. Digital identity management has become a central aspect of professional life for health care employees. In the digital era, professionals are required to navigate the complex boundaries between their personal and professional selves across various online platforms. Recent literature emphasizes that digital identity is dynamic and constructed through ongoing interactions, self-presentation, and audience perception [7]. Health care workers must balance authenticity, privacy, and professionalism, often engaging in “boundary work” to maintain appropriate conduct and protect sensitive information. This process involves critical self-awareness, digital literacy, and adherence to organizational and ethical guidelines for online behavior. Organizations are increasingly called upon to provide training and clear policies to support employees in managing their digital identities responsibly, thereby safeguarding both individual reputation and institutional trust [7,8].

### Nurses on Social Media

The public perception of nurses has frequently been influenced by media representations and stereotypes that do not accurately represent their knowledge or independence, which may have a detrimental effect on nurses’ professional identities and self-perception [9]. Nurses can use digital communities as a platform to present their public image and express their thoughts. They can criticize old assumptions and bolster their sense of professional worth by sharing their knowledge and achievements. Participation in an intraorganizational digital community can empower nurses by allowing them to acknowledge each other’s success and appreciate each other’s contribution.

Recent studies have emphasized the significance of such engagement. For instance, nurses are reported to play an essential role in encouraging knowledge exchange on digital health communities, and their social media confidence (knowledge self-efficacy) might significantly affect such conduct [10]. In other words, nursing leadership can stimulate greater involvement and knowledge sharing among their teams by actively facilitating dialogue on a digital community. According to the results of this study, the culture of knowledge sharing can be upgraded by having reliable nurses facilitate interactions in an intraorganizational Facebook group.

Nevertheless, nurses’ activities in a digital community raise a question that reveals a conflict between transparency and professional ethics. They must use their digital community activities responsibly for learning and support, enhancing their professional reputation, and increasing public awareness of their profession without breaking their professional boundaries or compromising other participants’ privacy [9]. This need can lead to setting standards and guidelines for what nurses can communicate on a digital media community. In reality, health care–providing organizations have social media use standards to guarantee adherence to ethical professionalism and discretion. These guidelines and nurses’ ethical standards shape an intraorganizational nurses’ community on Facebook [11].

This study aimed to examine the impact of an intraorganizational digital community on nurses’ professional identities and practices within a large health care organization. Specifically, the research aims to investigate how participation in a nurses-only intraorganizational Facebook community affects nurses’ sense of community, professional growth, and their daily practices.

## Methods

### Overview

This study used a qualitative descriptive approach to provide an extensive understanding of nurses’ experiences with an intraorganizational Facebook community. Participants who were most likely to contribute rich information pertinent to the research questions were chosen through a purposive sampling technique [12]. Being a registered nurse within the health care organization in any capacity, from management to staff nurse, and actively participating in the nurses-only intraorganizational digital community were prerequisites for inclusion. Participation was voluntary; ultimately, 10 nurses in managerial positions and 10 nonnurse managers were chosen (N=20). Hence, the sample represented diverse hospitals, units, and regions across the country, making it possible to record a variety of viewpoints. Recruitment continued until the researchers determined that data saturation had been accomplished, which means that no new themes or insights were revealed by further interviews [13].

### Participants

The study comprised 20 participants reflecting demographic and training diversity, highlighting the intraorganizational digital community’s varied influence on sense of community, professional growth, and burnout. Participants were aged between 33 and 62 years, married, and had children. Educationally, 2 (10%) held PhDs, 11 (55%) had master’s degrees, and 7 (35%) held bachelor’s degrees. A total of 10 (50%) participants held managerial positions. Participants came from diverse specialties, such as intensive care, palliative care, and psychiatry, with 14 (70%) working in hospitals and 6 (30%) in community clinics.

Including both nonmanagerial and managerial participant groups allowed us to explore how hierarchical position shapes experiences and interactions within the digital community. Notably, many (12/20, 60%) participants described how the usual boundaries between managerial and nonmanagerial staff were softened in the digital space, enabling open dialogue and mutual learning. The digital community often served as a social leveler, fostering egalitarian and supportive interactions that contrasted with traditional workplace hierarchies. This flattening of hierarchy aligns with the concept of “teaming” by Edmondson [14], which emphasizes the value of psychological safety and open communication across authority gradients to promote learning and innovation in organizations. However, some (8/20, 40%) participants remained aware of their hierarchical position, noting that concerns about professional image and credibility could influence their willingness to share openly, especially when managers were present in the discussions.

Only 3 (15%) participants had 10 or more years of experience in their current role, while 13 (65%) had over 20 years of nursing experience. This diversity helped capture a wide range of perspectives on the impact of participating in the digital community.

### Data Collection

After obtaining consent, individual interviews were conducted with each participant at times convenient for them, either in a private office or over a secure video connection, to address geographical distance. Each session lasted between 30 and 60 minutes. Using an interview guide, we asked open-ended questions about the nurse’s use of digital community (posting, commenting, reading discussions, etc), notable communications within the community, perceived advantages or disadvantages, and any effects on their work or professional views. We urged participants to offer concrete examples and probed further to thoroughly examine themes of interest. We tried to establish a relaxed atmosphere during the interview process, reminding nurses that their candid experiences were respected and that there were no right or wrong thoughts. To guarantee correct verbal information for analysis, the interviews were recorded and transcribed verbatim. Regarding confidentiality issues, any identifiable reference to a workplace, participants’ names, and other information that might identify specific patients, individuals, or complicated cases was either eliminated during transcription or substituted with aliases, general descriptions, or codes.

### Data Analysis

We examined the transcripts thematically, a structured qualitative approach for deriving and interpreting units of meaning, using aspects of content analysis to classify and condense informational material into themes and categories. Initially, 2 researchers individually reviewed all the transcripts several times to gain a thorough understanding of the data. Subsequently, they conducted an open coding process, analyzing the texts and labeling the concepts or ideas conveyed by the participants. Engaging in ongoing discussions and refinements, we extracted the data into common themes and categories representing the participants’ collective experiences. Thematic analysis followed the 6-phase reflexive approach by Braun and Clarke [15]. Coding reliability was ensured through continuous reflexive writing and peer debriefing with 2 experienced colleagues, who discussed and refined categories and themes. Three main themes emerged: sense of community, professional growth, and burnout, a theme not addressed in this paper.

### Ethical Considerations

This study was conducted in accordance with the ethical standards of the Declaration of Helsinki and received formal approval from the Helsinki Committee/Internal Review Board of the Meir Medical Center in Israel on August 13, 2024. All participants provided informed consent before participation, understanding the voluntary nature of their involvement and their right to withdraw at any time without consequences. Confidentiality and anonymity were strictly maintained throughout the research process. Data were securely stored, and any identifying information was removed or anonymized to protect participant privacy. The study adhered to the ethical guidelines recommended by the Journal of Medical Internet Research (JMIR) to ensure the integrity and ethical rigor of research involving human subjects and digital platforms.

## Results

This section presents the findings with sample quotes from the interviews to demonstrate each theme and its categories.

### Theme 1: Sense of Community

The intraorganizational digital community has emerged as a powerful source of emotional support, camaraderie, and professional belonging among nurses. Rather than functioning solely as an information exchange, participants described the community as a “digital family,” one that fosters deep interpersonal connections and mutual assistance. For example, a nurse recalled a moment of collective solidarity:

*When a colleague was injured in a road accident that also killed her sister, it was posted, and within an hour, hundreds of nurses expressed their sympathy and offered help…We never felt more united than at that moment.*

[P12]

This rapid mobilization of support illustrates how the digital platform enabled nurses to transcend geographical and organizational boundaries, reinforcing a shared identity and sense of unity. Many participants used family-related analogies, highlighting the intimacy and solidarity they experienced within the group. Another nurse shared the following:

*I won the hospital’s monthly excellence award…I received many heartfelt responses…It is a profound feeling that is hard to put into words, especially when all I did was hold my phone and cry.*

[P13]

Such expressions underscore the emotional resonance and validation provided by the community, extending beyond professional recognition to personal affirmation. The community also functioned as a social leveler, breaking down traditional hierarchical barriers. Nurses of all ranks interacted as peers, fostering egalitarian dialogue and collaborative problem-solving. One participant described this dynamic as follows:

*I surf at 2 AM with my first coffee and see a new idea from another ward…like learning from your sister at home how to help your children sleep.*

[P10]

This metaphor conveys both the informal, supportive atmosphere and the practical value of peer-to-peer learning. Importantly, the community was perceived as a safe space to vent frustrations, seek advice, or express feelings after a tough day, acting as an always-available peer support network—“much like an always-open extended nursing break room.”

However, some participants expressed concerns about authenticity, noting that the overwhelmingly positive tone sometimes inhibited open discussion of negative experiences or controversial viewpoints. As one nurse reflected:

*I was very pleased, but afterwards I got worried they would link me to posts, perhaps I would be perceived as too lightweight and not serious enough for roles with more responsibility.*

[P17]

This highlights the ongoing negotiation of professional image and impression management within the digital space [5]. Recent theoretical contributions further illuminate these dynamics—nurses in digital communities must continuously navigate issues of visibility, authenticity, and boundary work, balancing openness with the need to maintain a professional persona [8]. Thus, while the community fostered a strong sense of belonging and support, it also required members to manage their digital identities thoughtfully within organizational platforms.

### Theme 2: Professional Growth and Learning

Beyond emotional support, the digital community served as an informal, dynamic platform for professional development and learning. Nurses consistently reported that participation contributed to their knowledge, skills, and confidence in practice. Sharing knowledge was a central activity, exposing members to new cases, advice, and resources. One participant described the practical impact as follows:

*I am scrolling at 2 AM on my night shift when I have a minute for coffee, and I get new ideas I want to share with my supervisor.*

[P7]

This peer sharing complemented formal training and was perceived as more relevant because it originated from colleagues with direct experience. Such sharing exemplifies a “community of practice” [4], where rapid dissemination of knowledge enhances organizational performance [16]. Another nurse reflected on the tangible benefits:

*I felt that I genuinely knew more about emergency medicine, and that I would have the tools without having been taught formally how to react in real time.*

[P19]

Stories of professional initiative and success circulated within the community, inspiring others to innovate and take leadership. For example:

*I wrote a post about an innovative approach to patient guidance that we implemented, and the district chief executive nurse called me to say how proud she was.*

[P15]

Such recognition reinforced nurses’ professional identity and motivated further growth. The community also encouraged self-efficacy, particularly among younger nurses who felt empowered to ask questions and receive guidance from experienced colleagues. As one nurse shared:

*I never considered myself worthy of all this, but one post about a coffee shop I had established for our team…and today, I cannot go anywhere without people saying…I feel really recognized.*

[P16]

Importantly, participation in the digital community required nurses to navigate issues of visibility, authenticity, and professional boundaries in their learning and knowledge-sharing practices. As highlighted by recent theoretical contributions [8], nurses in organizational digital platforms must balance openness and vulnerability with the need to maintain a credible and professional online identity. This boundary work was evident as participants described both the empowerment and the caution that accompanied their engagement in the community.

Despite these benefits, participants acknowledged some limitations. The volume of daily posts sometimes made it difficult to keep up, and not all important information reached every member. However, most considered this a minor issue, as critical messages were also communicated through formal channels.

## Discussion

### Integrating the Themes: Community and Growth Hand-in-Hand

The 2 primary themes, professional growth and a sense of community, were closely connected when combined. Learning and growth processes were enhanced by the dynamic, collaborative, and interactive environment, and vice versa. Nurses were more inclined to share ideas, ask questions, and even acknowledge their lack of expertise because they felt safe and encouraged in the community (theme 1), which are vital behaviors for learning (theme 2). One nurse observed that while she might be reluctant to admit, “I do not know how to do X,” in a staff meeting, she witnessed colleagues in the digital community asking the same query openly, which motivated her to do the same without worrying about criticism. A sense of psychological safety characterizes effective learning communities. On the other hand, the community built ties while fostering nurses’ professional growth. For example, when a nurse embraced an idea posted on the community and reported success, it bolstered participants’ shared sense of improvement and fostered a sense of pride. In this respect, the community was an ideal setting for growth, and every case of progress enriched it.

Interestingly, our findings correspond to other innovative nursing training and practice endeavors. To help nursing students learn about mental health conditions, a recent study [17] used an interactive game-based application. The results indicated that the collaborative, peer-supported strategy improved students’ empathy for their peers and involvement. Similarly, our study found that the interactive, peer-driven digital community strengthened practicing nurses’ sensitivity to their peers and involvement in professional growth. Both studies found that although the settings differed (students learning through a game vs nurses learning through a digital community), the fundamental concept was the same: a team effort and emotionally supported learning produces more beneficial outcomes than top-down individualized approaches.

Additionally, our study addressed another pressing concern—nurses’ professional image and voice [9]. The intraorganizational digital community provided nurses with an enhanced organizational platform, a space to acknowledge achievements, communicate issues that worry them, and share pertinent thoughts and ideas. Such communication can address nurses’ internal professional identity.

The community constitutes an informal intraorganizational feedback venue, where nurses’ sense of worth develops with their awareness of certain managers participating in the digital community, attuned to their questions, expressing impressions regarding their suggestions and grievances, and possibly communicating supporting responses to their concerns much faster than in the formal arena.

### Implications for Practice

Despite a few drawbacks, this study yielded positive advantages of the intraorganizational digital community and introduced meaningful implications for health care organizations.

Organizations should consider supporting designated digital communities for staff to encourage and enhance intraorganizational communication and morale. Our study has shown that a nationwide, open, collegial online space can shatter boundaries between units, specialties, ranks, and geographic locations. This allows faster sharing of best practices and problems and a strong peer and organizational support network.

These findings suggest that nurse managers and leaders should actively engage in such forums and communities. A supportive leadership presence conveys the message that an organization values staff input. In our case, when leaders praised nurses or responded to advice-seeking, suggestions, shared success stories, worries, questions, and other communication within the community, it boosted nurses’ morale. Managers’ participation enabled the assessment of the level of staff concerns and interests in real time. An intraorganizational digital community can serve as grounds for initiating innovations and a preliminary warning system for identifying staff issues. The success of the digital community in this study gave nurses the sense that this was their space. Hence, although organizations can benefit from participation in the digital community, leaders should respect its informal nature and avoid top-down communication, using a semi-informal tone and engaging in equal participation.

In essence, organizations should embrace the compelling influence of a digital community. When nurses are willing to learn and contribute, they should focus their energy on promoting training opportunities, educational initiatives, and overall improvement efforts.

### Theoretical Implications

Theoretically, our findings reinforced and expanded several concepts in the literature. The digital community’s influence on professional identity corresponds to social identity theory [2]. Active participation reinforced nurses’ identification with fellow nurses and the organization. Regarding the community, nurses used the plural pronoun “we,” indicating a strong sense of cohesion. This may have affected their commitment to the organization and improved teamwork in their day-to-day practice, above and beyond the usual boundaries of their work units as a unified community, above and beyond their separate wards or clinics. This suggests that a collaborative and supportive community culture may outweigh conventional team-building practices.

Furthermore, our study clarified the role of digital professionalism, where nurses’ conduct in the digital community was ethical and professional. Participants were careful about what they posted, and the interviews yielded no unprofessional outbursts or privacy violations. This implies that nurses’ use of social media, especially in a closed, professional setting, was mature, which may result from growing knowledge regarding social media ethical protocol and its repercussions. Additionally, it implied that, in contrast to public social media, intraorganizational digital communities where users could be identified and held accountable for one another might generate more professional behavior. The significance of shared norms and community self-regulation could be emphasized in theoretical models of social media conduct in professional communities.

### Unanticipated Findings

Although we expected the digital community to contribute to organization-wide interactions, the degree to which traditional hierarchical barriers seemed to be dissolved within the community was a fairly surprising finding. Lower and higher rank nurses interacted on a first-name basis, occasionally resulting in increased openness to work. A staff nurse, for instance, said she felt she could easily and directly contact a nurse in charge regarding a problem because the latter had responded positively to a post she had shared. She might not have done that offline. Nonetheless, although it was not the focus of our study, the disintegration of hierarchy in the digital community was observed and is a noteworthy positive side effect.

Another unexpected finding that emerged from the interviews, although somehow linked to the previous one, is the pace at which the digital community culture influenced changes to day-to-day practice. Among others, following a heated group discussion concerning upgrading shift transfer, several units willingly explored a new shift-change routine. Another unit held a modest social gathering due to a post praising dedicated teamwork during a crisis. These instances demonstrated how the digital community actively changed the workplace atmosphere while also being reflected in it.

In summary, the examined extensive intraorganizational nurses’ digital community greatly impacted the nurses’ sense of community and professional identity. Active participation yielded stronger connections among nurses throughout the organization, creating a supportive environment that surpassed geographical boundaries and ward or expertise differences. This digital community emerged to strengthen a shared sense of community and pride among nurses and organization members.

The findings revealed that using the digital intraorganizational nurses’ community constituted a solid basis for strengthening nurses’ professional identity and mutual support and promoting ongoing professional learning and growth. Nurses shared knowledge, gained confidence, and strove to introduce new ideas to their practice. Giving staff a voice online impacted the nurses’ work lives, leading to long-term success and job satisfaction, which are crucial in the current health care climate.

Although limited in scope, examining 1 digital community in a single organization, albeit extensive, with a relatively small sample, and exploring only 2 aspects of nurses’ work, it is important to note that despite limited generalizability, the study yielded consistent and trustworthy findings. Future research will contribute to reaching a better and more profound picture by examining multiple organizations and diverse intraorganizational digital communities and using a mixed methods approach to assess the impact of digital communities on measurable professional and organizational results.

Although this qualitative study provides rich insights, several potential biases should be noted. Social desirability may have led participants to emphasize positive experiences or avoid criticism, especially given the professional context. Additionally, self-selection bias may have resulted in a sample that was more engaged or favorable toward the digital community. These factors may affect the credibility and transferability of the findings, despite efforts such as reflexive journaling and peer debriefing to enhance trustworthiness. Future research should include more diverse samples and triangulate data sources to address these limitations.

### Limitations

As a qualitative study, the findings reflect the subjective experiences and perspectives of a purposive sample within a single organization. Although this approach enables in-depth exploration and rich contextual understanding, it may limit the transferability of the results to other settings. Additionally, social desirability and self-selection biases may have influenced participation and responses, as those with strong opinions or positive experiences may have been more inclined to participate. Reflexive journaling and peer debriefing were used to enhance credibility, but these limitations are inherent to qualitative research. Future studies should consider broader sampling and triangulation across multiple organizations to deepen and validate these insights.

### Conclusions and Practical Implications

The findings of this study demonstrate that fostering a strong intraorganizational digital community constitutes a strategic investment in human capital. Such communities build relationships, facilitate knowledge sharing, and strengthen the collective professional identity of nurses. The evidence suggests that digital communities can serve as effective tools for organizational development; professional empowerment; and the creation of a unified, engaged nursing workforce.

### Policy and Training Recommendations

The following policy and training recommendations should be considered:

- Health care organizations should formally integrate digital communities into their communication and professional development strategies. These platforms enable rapid information flow, peer support, and collaborative problem-solving, which are essential for organizational resilience and adaptability [14,18].
- Training programs should include dedicated modules on digital professionalism and online identity management, equipping nurses and managers with the skills to navigate visibility, authenticity, and boundary work in digital environments [8].
- Leadership development should emphasize participation in digital communities as equals, fostering psychological safety and modeling constructive engagement.

By implementing these recommendations, health care organizations can leverage digital platforms to enhance staff well-being, morale, and professional growth, ultimately contributing to improved patient care and organizational outcomes.
